# Predictive model based on gene and laboratory data for intravenous immunoglobulin resistance in Kawasaki disease in a Chinese population

**DOI:** 10.1186/s12969-021-00582-6

**Published:** 2021-06-26

**Authors:** Li Meng, Zhen Zhen, Qian Jiang, Xiao-hui Li, Yue Yuan, Wei Yao, Ming-ming Zhang, Ai-jie Li, Lin Shi

**Affiliations:** 1 Capital Institute of Pediatrics-Peking University Teaching Hospital, Beijing, China; 2 Department of Cardiology, Children’s Hospital Capital Institute of Pediatrics, No. 2 Ya-Bao Road, Chao Yang District, Beijing, 100020 China; 3 Department of Cardiology, Beijing Children’s Hospital, Capital Medical University, National Center for Children’s Health, Beijing, China; 4 Department of Genetics, Capital Institute of Pediatrics, Beijing, China

**Keywords:** Kawasaki disease, Intravenous immunoglobulin resistance, Single nucleotide polymorphism

## Abstract

**Background:**

Here, we investigated the predictive efficiency of a newly developed model based on single nucleotide polymorphisms (SNPs) and laboratory data for intravenous immunoglobulin (IVIG) resistance in Kawasaki disease (KD) in a Chinese population.

**Methods:**

Data relating to children with KD were acquired from a single center between December 2015 and August 2019 and used to screen target SNPs. We then developed a predictive model of IVIG resistance using previous laboratory parameters. We then validated our model using data acquired from children with KD attending a second center between January and December 2019.

**Results:**

Analysis showed that rs10056474 GG, rs746994GG, rs76863441GT, rs16944 (CT/TT), and rs1143627 (CT/CC), increased the risk of IVIG-resistance in KD patients (odds ratio, OR > 1). The new predictive model, which combined SNP data with a previous model derived from laboratory data, significantly increased the area under the receiver-operator-characteristic curves (AUC) (0.832, 95% CI: 0.776-0.878 vs 0.793, 95%CI:0.734-0.844, *P* < 0.05) in the development dataset, and (0.820, 95% CI: 0.730-0.889 vs 0.749, 95% CI: 0.652-0.830, *P* < 0.05) in the validation dataset. The sensitivity and specificity of the new assay were 65.33% (95% CI: 53.5-76.0%) and 86.67% (95% CI: 80.2-91.7%) in the development dataset and 77.14% (95% CI: 59.9-89.6%) and 86.15% (95% CI: 75.3-93.5%) in the validation dataset.

**Conclusion:**

Analysis showed that rs10056474 and rs746994 in the *SMAD5* gene, rs76863441 in the *PLA2G7* gene, and rs16944 or rs1143627 in the interleukin (*IL)-1B* gene, were associated with IVIG resistant KD in a Chinese population. The new model combined SNPs with laboratory data and improved the predictve efficiency of IVIG-resistant KD.

**Supplementary Information:**

The online version contains supplementary material available at 10.1186/s12969-021-00582-6.

## Background

Kawasaki disease (KD) is an acute vasculitis of unknown etiology that predominantly occurs in children under 5 years of age. The major complication of KD is coronary artery lesions (CALs) [1], including coronary artery dilatation and coronary aneurysm (CAA); these may subsequently result in long-term sequelae such as coronary stenosis, coronary obstruction, and myocardial infarction. KD has become the leading cause of acquired childhood heart disease in developed countries [2].

A single high dose of intravenous immunoglobulin (IVIG), together with a high dose of acetylsalicylic acid (ASA) is the current standard treatment for acute KD and can effectively reduce CAA from 20 to 25% to 3-5% [3]. However, approximately 10-20% of patients still experience persistent or recurrent fever after standard treatment,which defines IVIG-resistance [4, 5]; Patients not responding to IVIG are at heightened risk for CALs [6]. Therefore, there is an urgent need to develop methods to predict the IVIG therapeutic response before initial treatment in order to improve prognosis by individualized treatment. Several scoring systems comprising clinical features and laboratory data have been proposed to predict IVIG resistant in KD patients representing various geographic locations and ethnicities with good sensitivity and specificity for the respective source populations [7–11]. However, these systems are not sufficient for other populations [12], thus indicating that IVIG resistance might be related to genetic and ethnic factors. Previous genome-wide association studies (GWASs) have demonstrated a correlation between genetic factors and IVIG resistant KD in different ethnic populations [13, 14]. Therefore, the present study was designed to investigate the genes associated with IVIG resistance in a Chinese population and investigate whether combining this genetic information with the existing scoring system could improve the predictive efficacy.

## Methods

### Patients

We recruited children with KD who had been hospitalized in the cardiology department at the Children’s Hospital Capital Institute of Pediatrics between December 2015 and August 2019. Data acquired from this cohort (the development dataset) were then used to screen target genes and construct the predictive model. Data from a second cohort (the validation dataset) were acquired from children with KD who had been hospitalized at the Beijing Children’s Hospital were then used to validate the model. Our enrollment time was after receiving the first dose of IVIG and treatment failure.

The diagnosis of KD was based on the guidelines proposed by the American Heart Association in 2004 [15], and parents or guardians provided informed consent to participate in the study. Children were diagnosed with KD if they had experienced fever for at least 5 days and fulfilled four or more of the five major clinical features of KD (erythema of the mucosa with a strawberry tongue and cracked lips, bilateral non-purulent conjunctivitis, cervical lymphadenopathy, polymorphous rash, and swelling or redness of the extremities in the acute phase or periungual desquamation in the subacute phase). However, patients who had experienced fever for ≥5 days and had < 4 of the principal features were also diagnosed with KD if coronary aneurysm or dilatation was evident on 2-dimensional echocardiography or coronary angiography. After the diagnosis of KD, all patients were treated with a high dose of IVIG (2 g/kg) and a high dose of aspirin (30-50 mg/kg.d, Tid); these drugs were taken orally within 10 days of disease onset. Patients were excluded from the study if they had comorbidity with another rheumatic or infectious vasculitis, or contraindication to IVIG (i.e., an allergy to IVIG), or if they had an incomplete clinical dataset for the predictive model.

Patients who had experienced fever for at least 5 days and fulfilled the criteria for four or five principal clinical features, or had four principal clinical features along with coronary artery abnormalities on echocardiography, were diagnosed with complete KD (cKD) [15]. Patients who had three principal clinical features, with coronary artery abnormalities on echocardiography, but were devoid of other febrile illnesses were defined as incomplete KD (iKD). Patients who had experienced persistent fever for 36 h (an axillary temperature > 38 °C) after the initial IVIG infusion, or had experienced recrudescent fever within 7 days of IVIG treatment accompanied by at least one of the clinical manifestations of KD except for other reasons, were defined as IVIG-resistant [15]. Due to the costs involved in genetic data analyses, we utilized matching methods in this study. Patients who met the criteria were IVIG-resistance were recruited as an IVIG-resistance group. We also recruited an age- and sex-matched IVIG-response group during the same period of hospitalization (in a 1:2 ratio).

This study was approved by the Ethics Committee of the Capital Institute of Pediatrics (Reference: SHERLL 2015040). Informed consent was obtained from a parent or guardian of each patient prior to the study.

### Target gene selection and genotype detection

First, we retrieved literature published before 2015 from the human genome retrieval website (https://www.ncbi.nlm.nih.gov/) provided by the NCBI, and selected 18 SNPs that may be associated with IVIG resistance in patients with KD [16–24]. (Supplementary Table 1) We also evaluated the distribution of these SNPs in healthy East Asian populations using the human genome retrieval website. (Supplementary Table 2).

From each patient, we acquired a 2 ml sample of peripheral blood in an EDTA anticoagulant tube following early morning fasting and after the diagnosis of KD but before the initiation of treatment (during the early stages of disease; 6-10 days from the onset of fever). Serum was separated by centrifugation and 200-500 μg of blood cells were stored in a refrigerator at − 80°c for DNA extraction. The genotypes of the 18 SNPs were determined by Sanger sequencing.

DNA extraction and PCR procedures followed the standard operating procedures for one generation sequencing (ABI 3730xl, USA). Sequencing results were compared with a reference sequence using Mutation Surveyor v4.0 software (Softgenetics, USA).

The quality control procedures were carried out in the following steps. A sample of each genotype was randomly selected for DNA sequencing to verify the allele sequence; results were 100% concordant to the initial analysis. All images acquired from the agarose gel electrophoresis of PCR amplicons,and absolute quantification curves for fluorescence data derived from Taqman assays, were conducted by an independent researcher. Furthermore, 5% of samples were randomly selected and run in duplicate to ensure the accuracy of the genotyping.

### Clinical data

We collected a range of clinical data, including age, sex, and the laboratory indicators involved in the predictive model established previously, such as the percentage of neutrophils (N%), C reactive protein (CRP), albumin (ALB), sodium ion concentration (Na), and total bilirubin (TBIL). If these laboratory data were performed several times before treatment, the highest value was adopted for N%, CRP, and TBIL, while the lowest value was adopted for ALB and Na.

### Statistical analysis

Our research team included a pediatrician and a data analyst. When the patients were admitted, the pediatrician made a diagnosis according to specific diagnostic criteria; the data were collected and analyzed by the data analyst. The outcome assessors were blinded to the groups that the patients had been assigned to.

We used a range of statistical software packages to analyze our data, including SPSS (Version 25.0), R (Version 3.6.0), Haploview (Version 4.2), and MedCalc 11.4.2.0. Data were expressed as Mean ± standard deviation (SD) for continuous variables or a percentage of the total number of patients for categorical variables. The Student’s t-test was used to compare continuous variables and the chi-square test was used to compare categorical data. *P* < 0.05 was considered to be statistically significant.

SNP association analysis refers to the non-random association of different alleles that are combined as a small module to influence expression; LD gene association analysis is the predominant method used at present [25]. Hardy-Weinberg equilibrium (HWE) analysis was performed for each SNP to determine deviations of the observed genotypic distribution from the expected distribution; this information was analyzed by Haploview software. The genotypic frequency of SNPs was compared using univariable and multivariable logistic regression analysis. SNPs that exhibited a *P* < 0.01 in the univariate analysis were then included in multivariable analysis as covariates to output SNP loci that could influence the responsiveness of IVIG. In addition, multivariable logistic regression was used to analyze correlations between the genotypes of the SNPs and the risk of IVIG-resistance by adjusting confounders, such as gender or age. We also determined the crude odds ratio (Crude OR), adjusted OR (AOR), and 95% confidence interval (CI).

The established model featured several key laboratory variables [26]. Numerical variables used in the previous model were turned into dichotomous variables by adopting appropriate cut-off points for logistic regression. The SNPs used in the Nomogram were then turned into dichotomous variables according to the type of mutation (wild type vs mutant). Each variable was then assigned score points according to the Nomogram, and the total number of points was calculated for each patient. The final risk score was divided into two risk strata (low-risk or high-risk group). The predictive power of different models was evaluated by creating receiver operator characteristic curves (ROC). The method described by DeLong et al. [27] was then used to compare the area under the ROC curves (AUC) between the new model and the previous models. The goodness-of-fit of the model was evaluated by the Hosmer-Lemeshow test [28]; *P* > 0.05 indicated a lack of deviation between the model and the observed event rate.

## Results

### Demographic data

**Fig. 1:**
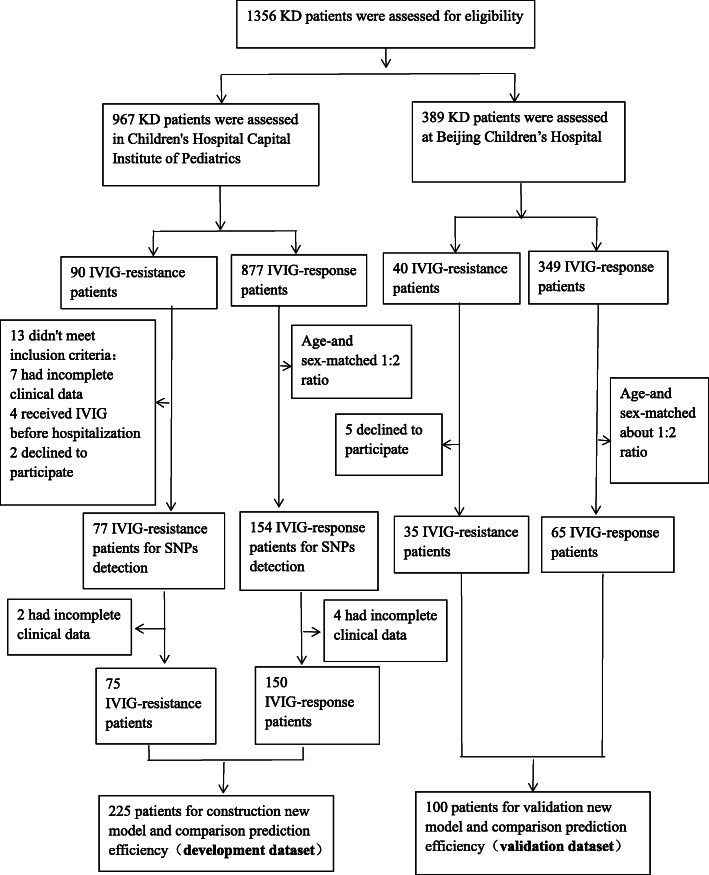
Flow chart of enrolled participants

A total of 331 children with KD were enrolled into this study (Fig. 1). In total, 231 cases were enrolled in the development dataset: 77 patients (male/female = 61/16, mean age: 2.28 ± 1.56 years) were placed in the IVIG-resistance group and 154 patients (male/female = 120/34, mean age: 2.31 ± 1.54 years) were placed in the IVIG-response group. A further 100 patients were enrolled in the validation dataset: 35 patients (male/female = 25/10, mean age: 2.51 ± 2.06 years) were placed in the IVIG-resistance group and 65 patients (male/female = 45/20, mean age:1.95 ± 1.65 years) were placed in the IVIG-response group (Table 1).

**Table 1:** General clinical data of KD children

	IVIG-resistance	IVIG-response	*P*-value
Age (Year)(X ± S)	2.28 ± 1.56	2.31 ± 1.54	0.92
≤ 1	25 (32.5%)	36 (23.4%)	0.34
1-5	48 (62.3%)	109 (70.8%)	
≥ 5	4 (5.2%)	9 (5.8%)	
Sex (Male/Female)	61/16 (3.81:1)	120/34 (3.53:1)	0.82

*IVIG* intravenous immunoglobulin, *KD* Kawasaki disease

### LD and HWE analysis

**Fig. 2:**
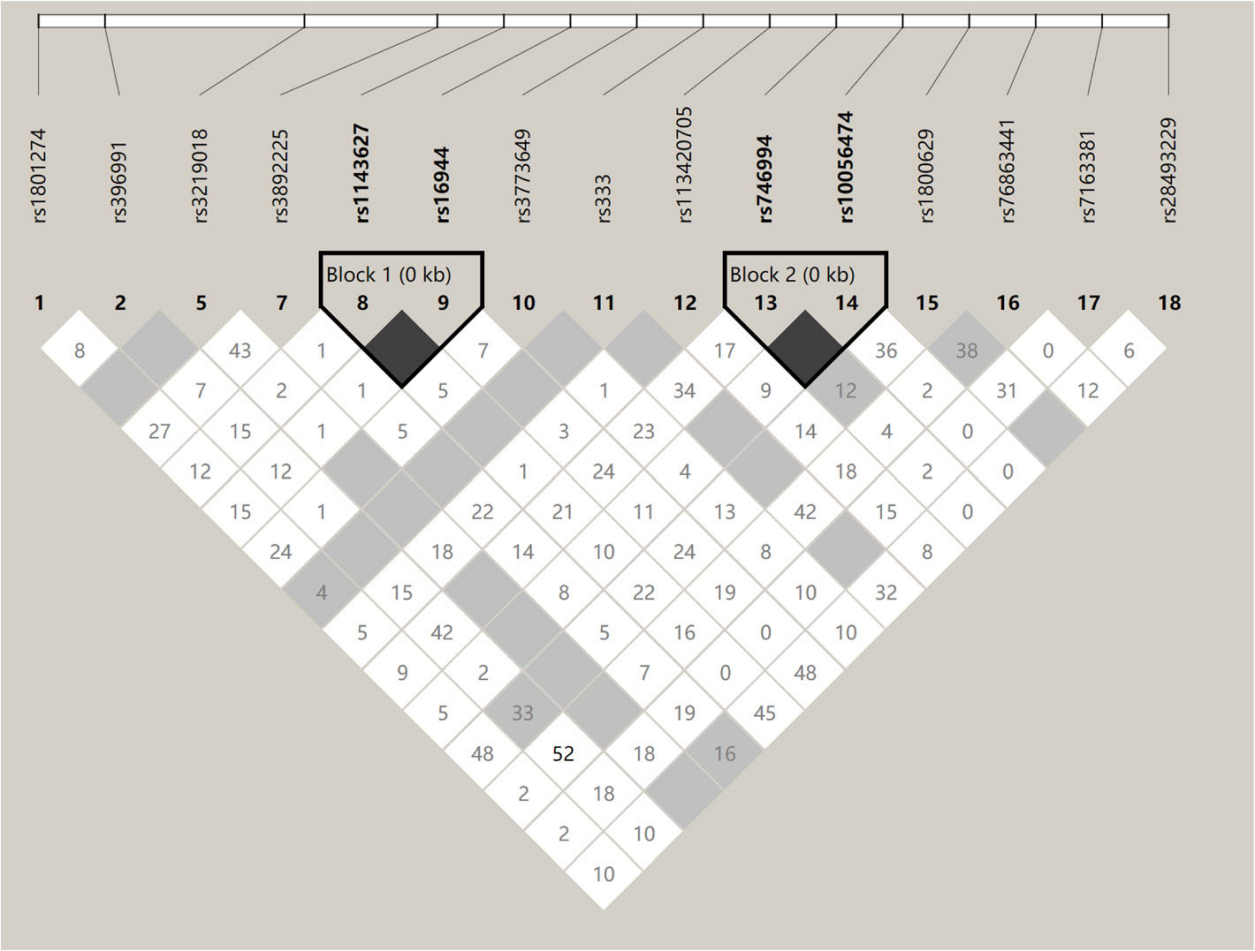
Results of linkage disequilibrium analysis

Haploview was used to analyze the 18 identified SNPs, as shown in Fig. 2. This analysis revealed that the rs16944 and rs1143627 loci in the interleukin (IL)-1B gene were in complete LD (D′ = 1.0, *r*^2^ = 0.974) and that the rs10056474 and rs746994 loci in the *SMAD5* gene were in incomplete LD (D′ = 1.0, *r*^2^ = 0.222). Further analysis revealed that the rs403016 and rs447536 loci were not in HWE (*P* < 0.001). Some mutations were detected in rs3219018, rs780467580, and rs333, with a minor allele frequency (MAF) < 0.01 [29]).

### Logistic regression analysis of high-risk SNPs for IVIG-resistance

According to LD and HWE analysis, rs3219018, rs780467580, and rs333, loci were excluded due to the presence or absence of rare mutations (MAF < 0.01). We also excluded rs403016 and rs447536 loci owing to the fact that these were not in HWE. After adjusting for these factors, a total of 13 SNPs were used for univariate analysis.

Furthermore, a comparison of genotype frequencies showed that cases of KD with a *SMAD5*-rs10056474 GG genotype had a 2.459-fold higher risk of IVIG-resistance than those with CC/CG genotypes (OR, 2.459; 95%CI, 1.185-5.101) (Table 2).

**Table 2:**
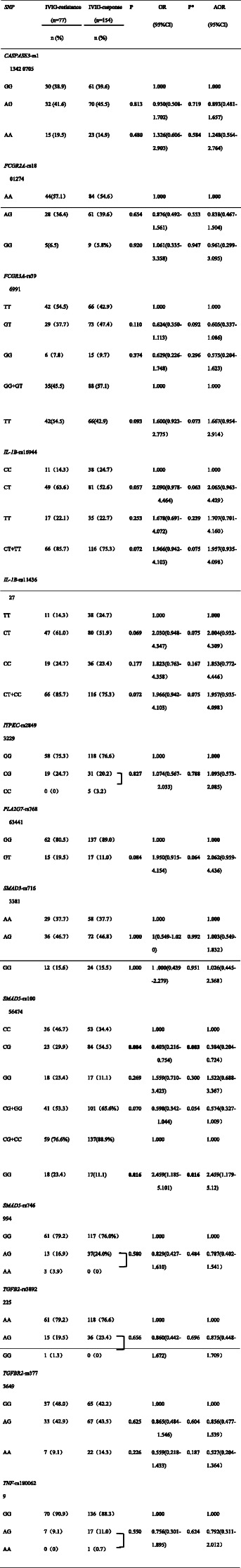
Comparison of distribution of 13 SNPs genotypes between IVIG-resistance and IVIG-response group

*IVIG* intravenous immunoglobulin, *KD* Kawasaki disease, *OR* odds ratio, *AOR* Adjusted odds ratio. AOR and P* were adjusted for gender and age (≤1,>1 and < 5, ≥5)

SNPs that exhibited a *P* < 0.01 in the univariate analysis (rs10056474, rs76863441, rs16944, rs114362, and rs396991) were then included in multivariable analysis. rs746944 was included as an interactive variable for rs10056474 due to incomplete LD (D′ = 1.0, *r*^2^ = 0.222). Furthermore, because there was a complete LD between rs16944 and rs1143627 (D′ = 1.0, *r*^2^ = 0.974), neither rs16944 or rs1143627 was included. Finally, 5 SNPs (rs10056474, rs746994, rs76863441, rs16944 (or rs1143627), and rs396991) were included in the multivariable logistic analysis. Results showed that the rs10056474 and rs746994 loci of the *SMAD5* gene, the rs76863441 locus of the *PLA2G7* gene, and the rs16944 or rs1143627 locus of the *IL-1B* gene, were correlated with IVIG-resistance in children with KD. (Table 3).

**Table 3:** Multivariable regression analysis of IVIG-resistance of KD

Variables	*P*	OR	95%CI	
			Lower	Upper
rs76863441 GT	0.061	2.097	0.968	4.542
rs10056474 GG by rs746994 GG	**0.013**	3.723	1.317	10.524
rs16944 (CT/TT) or rs1143627(CT/CC)	0.054	2.107	0.987	4.494
Constant	0.000	0.220		

*IVIG* intravenous immunoglobulin, *KD* Kawasaki disease, *OR* odds ratio, *CI* confidence interval

### A predictive model for IVIG-resistance

**Fig. 3:**
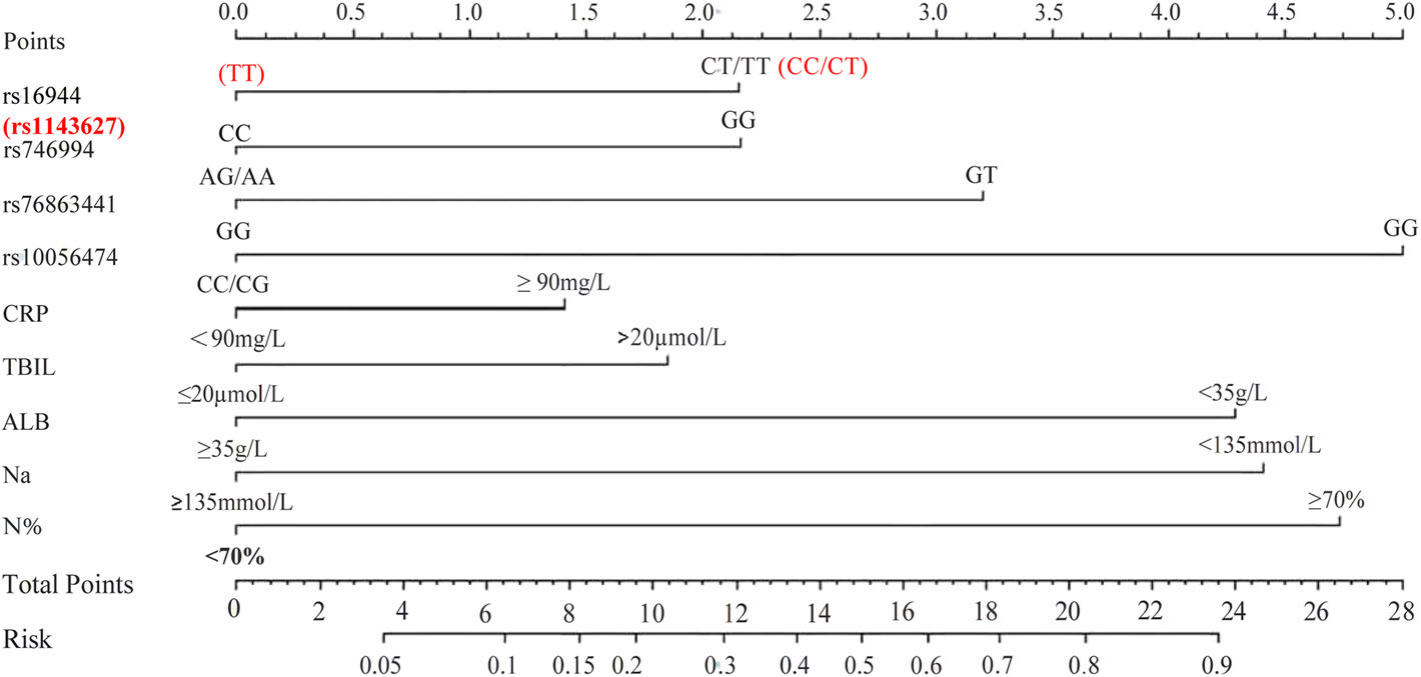
Nomogram. (rs16944 CT/TT: 2 points (rs1143627 CC/CT: 2 points); rs746994 GG: 2 points; rs76863441 GT: 3 points; rs10056474 GG: 5 points; CRP ≥ 90 mg/L: 1.5 points; TBIL> 20 μmol/L: 2 points; ALB< 35 g/L: 4.5 points; Na < 135 mmol/L: 4.5points; N% ≥70%: 5 points)

In the present study, five SNPs were found to be associated with IVIG-resistance. These SNPs, along with the laboratory indicators used in the previous model [26] (Table 4) were used as variables to create a new model. In total, data from 225 KD patients (75 IVIG-resistance, 150 IVIG-response) were used to construct a new predictive model; 6 patients had been excluded due to incomplete laboratory data. Each variable was scored using a Nomogram (Fig. 3). A total score > 12.5 points was considered high-risk for IVIG resistance; the AUC was 0.832 (95%CI: 0.776-0.878) and the sensitivity and specificity were 65.33% (95%CI:53.5-76.0%) and 86.67% (95%CI:80.2-91.7%), respectively. There was no significance with regards to the Hosmer-Lemeshow statistic (*P* = 0.585 > 0.05).

**Table 4:** Old model used to predict IVIG resistance in patients with KD [26]

Variables	Points	Predicted risk (score)
CRP ≥ 90 mg/L	3	High risk (**≧6** points) Low risk (0-5 points)
N% ≥ 70%	2.5	
Na<135 mmol/L	3	
ALB<35 g/L	2.5	
TBIL>20 μmol/L	5	

*CRP* C reactive protein, *N%* Percentage of neutrophils, *Na* Sodium ion concentration, *ALB* albumin, *TBIL* total bilirubin

### Validation and comparison of the model

Data from patients hospitalized at Beijing Children’s Hospital were then used to validate the newly constructed model. The AUC was 0.820 (95% CI: 0.730-0.889) while the sensitivity and specificity were 77.14% (95% CI: 59.9-89.6%) and 86.15% (95% CI: 75.3-93.5%), respectively.

**Fig. 4:**
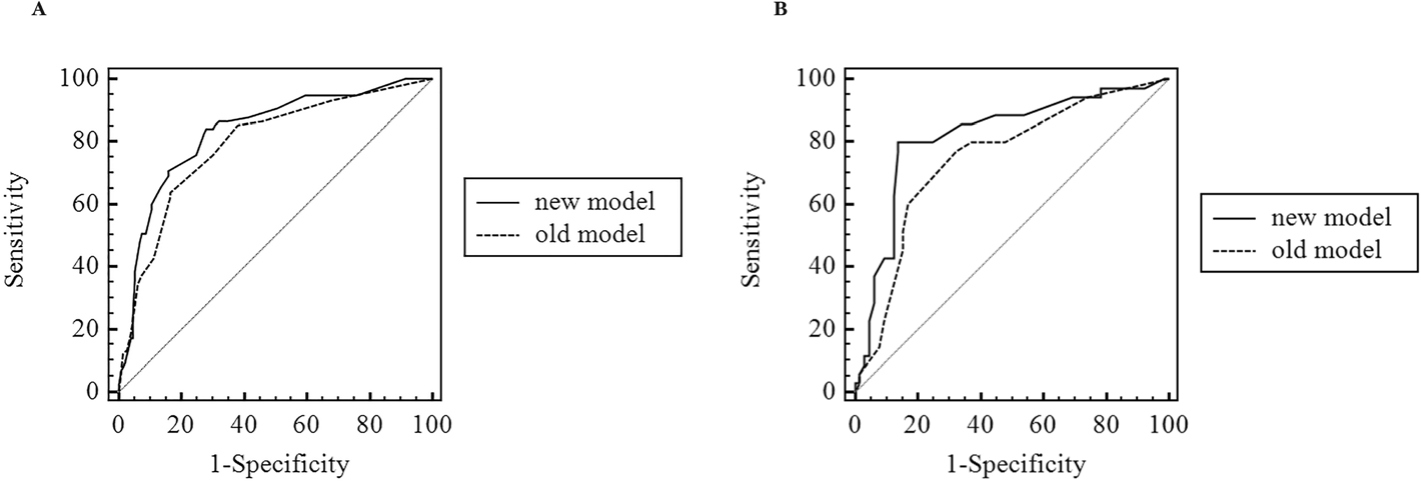
ROC curves of the two models. (**A**: ROC curves in development dataset; **B**: ROC curves in validation dataset)

Comparisons of the new and previously established. Model showed that the AUC was higher than the previous model in the development dataset (0.832, 95% CI: 0.776-0.878 vs 0.793, 95% CI: 0.734-0.844, Z = 2.316, *P* = 0.021) and in the validation dataset (0.820, 95% CI: 0.730-0.889 vs 0.749, 95% CI: 0.652-0.830; Z = 2.145, *P* = 0.032) (Fig. 4).

## Discussion

The present study showed that SNPs in the rs10056474 and rs746994 loci of the *SMAD5* gene, in the rs76863441 loci of the *PLA2G7* gene, and the rs16944 or rs1143627 loci in the *IL-1B* gene, were all associated with IVIG-resistance in children with KD in a Chinese population. The SNPs of these genes can be sequenced quickly by the Sanger method, which is convenient and cheap for clinical application. The results of Sanger sequencing to identify the SNPs in the text can be completed in our laboratory, and it takes about 4 h from specimen collection to experimental results. Moreover, the new predictive model, based on gene polymorphisms and laboratory data, showed improved predictive efficacy for IVIG-resistance and may help pediatricians to identify high-risk IVIG-resistant patients prior to the initiation of treatment in order to improve the prognosis of patients with KD [30]. With regards to the high-risk patients with IVIG resistance that were selected by the predictive model, we preferred to recommend the addition of corticosteroids or infliximab into their treatment plans; this strategy was based on the 2017 AHA scientific statement and clinical practice in our center [4]. Other alternative adjunctive therapies, such as cyclosporine, cyclophosphamide, and plasma exchange, have been recommended in the literature [4].

.The pathogenesis of IVIG-resistance in children with KD has yet to be fully elucidated. Numerous studies have reported that IVIG-resistant KD is associated with genetic background. However, most of the studies that were reported previously focused on one gene or several genes within a particular pathway in different populations; few studies investigated multiple genes within the same population [31]. Studies have also shown that multiple gene associations may be of greater predictive value than a single gene association [32]. In this study, we selected multiple genes that were possibly related to IVIG-resistance that had been reported in different ethnic populations. We used these to evaluate the role of gene polymorphisms in the therapeutic response to IVIG in a Chinese population.

This study showed that two loci (rs10056474 and rs746994) in the *SMAD5* gene were associated with IVIG-resistance in children with KD. rs10056474 GG was assigned the highest score in the predictive model; this may indicate that the *SMAD5* gene plays an important role in IVIG-resistance. *SMAD5*, as an intracellular mediator of the transforming growth factor (TGF)-β signal transduction pathway, is involved in the induction of angiogenesis, cardiomyocyte hypertrophy, calcification, and fibrosis in the cardiovascular system [33]. Several studies have shown that the SMAD protein family is correlated with KD susceptibility and therapeutic response; however, these studies mainly focused on the *SMAD3* and *SMAD4* genes [23]. Some previous studies on *SMAD5* gene polymorphisms in KD demonstrated that these variations were associated with KD susceptibility rather than IVIG therapeutic response [34]. The rs10056474 and rs746994 loci of the *SMAD5* gene were related to IVIG-resistance and located in the introns of the *SMAD5* gene. This may be associated with the fact that *SMAD5* can be activated by bone morphology proteins (BMPs) in the TGF-β pathway to participate in angiogenesis. However, our present study did not find that any of the genes in the TGF-β pathway, except for *SMAD5*, were associated with IVIG-resistance; these findings are consistent with a previous study carried out in Taiwan [35].

.The rs76863441 loci of the *PLA2G7* gene were associated with IVIG-resistant KD in this study. Platelet-activating factor acetylhydrolase (PAF-AH), also known as lipoprotein-associated phospholipase A2 (Lp-PLA2), is encoded by the *PLA2G7* gene and maintained stable by PAF (a form of inflammatory factor) at appropriate levels to play an anti-inflammatory role [36]. Previous studies have shown that *PLA2G7* gene polymorphisms determine plasma PAF-AH activity [37]. In the present study, we found that IVIG resistance in the rs76863441GT genotype was significantly higher than that in the rs76863441GG genotype; this was consistent with previous observations reported by Minami et al. which stated that IVIG-resistance in the GT + TT genotype was significantly higher than that in the GG genotype [22]. When the G base is replaced by the T base, resulting in a change in the genetic code from GUU to UUU, and substitution of valine (Val) with phenylalanine (Phe) at position 279, there was a clear reduction in PAF-AH activity; subsequent increases in the level of PAF level, and the activation of production for other inflammatory mediators [38], may underlie the poor therapeutic response to IVIG in KD.

The two SNPs (rs16944, rs114362) in the *IL-1B* gene were found to be related to the therapeutic response to IVIG in this study. The resistance of IVIG in the rs16944CT/TT genotype was significantly higher than that in the CC genotype and the resistance of IVIG in the rs1143627CT/CC genotype was significantly higher than that in the TT genotype; these findings were consistent with those reported previously by Taiwanese scholars, who stated that the rs16944TT and rs1143627CC genotypes were associated with a significant increase in the risk of IVIG resistance [21]. We also found that the rs16944 and rs1143627 SNPs had strong LD, as observed previously [39]; consequently, either rs16944 or rs1143627 could exhibit biological activity at these two loci. IL-1B is an inflammatory cytokine produced by activated macrophages and plays a key role in the pathogenesis of KD. Both the rs16944 and rs11436227 SNPs were located in the promoter region of *IL-1B* which regulates the transcription of IL-1B. Children with the rs16944CT/TT or rs1143627CT/CC genotype may exhibit increased IL-1B levels; this gives rise to the chemotaxis of leukocytes and the induction of neutrophils to release large amounts of cytokines, thus resulting in a poor response to IVIG treatment [40]. A case report on the efficacy of an IL-1 receptor antagonist (Anakinra) on IVIG-resistant KD patients [41], along with ongoing clinical trials involving the blockade of IL-1 or the treatment of acute KD [42], further confirmed this conjecture.

There are some limitations to this study that need to be considered. First, because of the high costs associated with SNP testing, we used a ratio of 1:2 to match IVIG-resistant versus IVIG-responsive cases. Thus, 85.6% (77/90) of IVIG-resistant cases and 17.6% (154/877) of IVIG-responsive cases were included. The much lower proportion of IVIG responsive patients may have led to selection bias. However, the general characteristics between the matched and non-matched children in the response population were compared and there was no statistical difference (Supplementary Table 3). Second, although the sample size for the development model was small and derived from a single center, the center involved is one of the largest children’s hospitals in the Beijing area and patients attend this hospital from all over the country. In the present study, 90% of the patients were from northern China while 10% were from southern China; they were all Han Chinese. More importantly, the quality of our study was guaranteed by the same brand of IVIG that was used in the center; the treatment response was observed by the same researchers in accordance with consistent standards. Third, although we validated our model at another center with good results, it is possible that there may be issues related to external validity or generalizability. Furthermore, our study population only featured Chinese patients; whether the results of this study are applicable to other ethnicities needs further investigation and validation. Overall, our findings need to be validated in a larger dataset in future. Finally, our patients were recruited from the two largest children’s hospitals in the Beijing area in which the children have a more serious condition. Consequently, our prediction model may only be applicable to medical institutions that are similar to these two children’s hospitals.

In summary, rs10056474 and rs746994 SNPs in the *SMAD5* gene, the rs76863441 SNP in the *PLA2G7* gene, and the rs16944 or rs1143627 SNPs in the *IL-1B* gene, were all associated with IVIG resistant KD in the Chinese population. Our new model combines SNPs with laboratory data and improved the prediction efficiency of IVIG-resistant KD. However, whether our model can be generalized to other ethnic populations requires further study.

## Supplementary Information

**Additional file 1.**

## Data Availability

All data generated or analyzed during this study are included in this published article and its supplementary files.

## Abbreviations

AHA: American Heart Association
ALB: Albumin
AOR: Adjusted odds ratio
ASA: Acetysalicylic acid
AUC: Area under the curve
BMPs: Bone morphology proteins
CALs: Coronary artery lesions
CAA: Coronary aneurysm
CI: Confidence interval
c-KD: Complete Kawasaki disease
CRP: C-Reactive protein
GWAS: Genome-wide association study
HWE: Hardy-Weinberg equilibrium
IL: Interleukin
iKD: Incomplete Kawasaki disease
IVIG: Intravenous immunoglobulin
KD: Kawasaki disease
LD: Linkage disequilibrium
Lp –PLA: Lipoprotein-associated phospholipase A
Na: Sodium ion concentration
OR: Odds ratio
PAF-AH: Platelet-activating factor acetylhydrolase
ROC: Receiver operator characteristic
SNP: Single nucleotide polymorphism
TBIL: Total bilirubin
TGF: Transforming Growth Factor
