# Supplementary material for: Predictive model based on gene and laboratory data for intravenous immunoglobulin resistance in Kawasaki disease in a Chinese population
Source: Pediatr Rheumatol Online J. 2021 Jun 26;19:95. doi: 10.1186/s12969-021-00582-6 (PMC8236184; doi:10.1186/s12969-021-00582-6)
Supplement: Supplementary file 1 — Additional file 1. [file 12969_2021_582_MOESM1_ESM.docx]

**Supplementary Table 1** 18 SNPs associated with IVIG-resistance in KD

| Gene | SNP | Allele | Reference | Population |
| --- | --- | --- | --- | --- |
| CCR5 | rs333 | WW/W-* | [16] | East Asian(Japanese) |
| CASPASE3 | rs113420705 | G/A | [17] | East Asian(Japanese) |
| FCGR2B | rs3219018 | G/C | [18] | Northwestern United States |
| FCGR2B | rs780467580 | T/A | [18] | Northwestern United States |
| FCGR2A | rs1801274 | A/G | [19] | European and Asian |
| FCGR3A | rs396991 | T/G | [20] | European and Asian descent |
| FCGR3B | rs403016 | C/G | [20] | European and Asian descent |
| FCGR3B | rs447536 | C/T | [20] | European and Asian descent |
| IL-1B | rs16944 | C/T | [21] | East Asian ( Taiwanese) |
| IL-1B | rs1143627 | T/C | [21] | East Asian ( Taiwanese) |
| ITPKC | rs28493229 | G/C | [17] | East Asian(Japanese) |
| PLA2G7 | rs76863441 | G/T | [22] | East Asian(Japanese) |
| SMAD3 | rs7163381 | A/G | [23] | American |
| SMAD5 | rs10056474 | C/G | [23] | American |
| SMAD5 | rs746994 | G/A | [23] | American |
| TGFB2 | rs3892225 | A/G | [23] | American |
| TGFBR2 | rs3773649 | G/A | [23] | American |
| TNF-α | rs1800629 | G/A | [24] | East Asian(Chinese) |

**Supplementary Table 2** Distribution frequency of 18 SNPs alleles in the general population of East Asian

| Gene | SNP | Allele | Allele (N%) | |
| --- | --- | --- | --- | --- |
|  |  |  | Ref Allele | Alt Allele |
| CCR5 | rs333 |  | WW (100.00%) | W-* (0.00%) |
| CASPASE3 | rs113420705 |  | A (34.30%) | G (65.70%) |
| FCGR2B | rs3219018 |  | G (80.00%) | C (20.00%) |
| FCGR2B | rs780467580 |  | T (100.00%) | A (0.00%) |
| FCGR2A | rs1801274 |  | A (64.93%) | G (35.07%) |
| FCGR3A | rs396991 |  | T (67.52%) | G (32.48%) |
| FCGR3B | rs403016 |  | C (99.1%) | G (0.90%) |
| FCGR3B | rs447536 |  | C (99.1%) | T (0.90%) |
| IL-1B | rs16944 |  | T (44.91%) | C (55.09%) |
| IL-1B | rs1143627 |  | C (45.66%) | T (54.34%) |
| ITPKC | rs28493229 |  | G (93.71%%) | C (6.29%) |
| PLA2G7 | rs76863441 |  | G (96.41%) | T (3.59%) |
| SMAD3 | rs7163381 |  | A (64.94%) | G (35.06%) |
| SMAD5 | rs10056474 |  | C (62.21%) | G (37.79%) |
| SMAD5 | rs746994 |  | G (89.42%) | A (10.58%) |
| TGFB2 | rs3892225 |  | A (81.34%) | G (18.66%) |
| TGFBR2 | rs3773649 |  | G (69.92%) | A (30.08%) |
| TNF-α | rs1800629 |  | G (91.10%) | A (8.90%) |

*：WW:ACAGTCAGTATCAATTCTGGAAGAATTTCCAGACA；—:ACA ；CCR5:C-C Chemokine Receptor type 5; CASPASE-3:Cysteinyl Aspartate Specific Proteinase-3; FCGR:Fcgamma receptor; IL-1B:Interleuki-1beta;ITPKC:Inositol 1,4,5-Triphosphate 3-Kinase C;PLA2G7:Platelet-Activating Factor Acetylhydrolase;SMAD:Drosophila mothers against decapentaplegic protein;TGFB:Transforming Growth Factor beta;TGFBR:Transforming Growth Factor beta Receptor;TNF-α:Tumor Necrosis factor-α.

**Supplementary Table 3** General characteristics between matched and non-matched children in IVIG-responsive Children with KD

| Variables | Building model（N=877） | | P | Validation model（N=349） | | P |
| --- | --- | --- | --- | --- | --- | --- |
|  | Matched children  (N=154)  （n,%） | Non-matched children  (N=723)  (n,%) |  | Matched children  （N=65)  (n,%) | Non-matched children  (N=284)  (n,%) |  |
| Male | 120 (77.9%) | 545(75.4%) |  | 45 (69.2%) | 205(72.2%) |  |
| Female | 34(22.1%) | 178 (24.6%) | 0.50 | 20(30.8%) | 79(27.8%) | 0.63 |
| iKD | 3 (1.9%) | 10 (1.4%) |  | 2(3.1%) | 4(1.4%) |  |
| cKD | 151(98.1%) | 713(98.6%) | 0.60 | 63(96.9%) | 280(98.6%) | 0.35 |
| Age (Years) | 2.31±1.54 | 2.51±1.70 | 0.18 | 1.95±1.65 | 2.18±1.72 | 0.33 |

KD: Kawasaki disease; iKD: Incomplete KD；c-KD: Complete KD; IVIG:

Intravenous Immunoglobulin.
